# Draft Genome Sequences of Megaplasmid-Bearing Streptomyces sp. Strains BF-3 and 4F, Isolated from the Great Salt Plains of Oklahoma

**DOI:** 10.1128/genomeA.00208-18

**Published:** 2018-04-05

**Authors:** Carolyn R. Cornell, Daya Marasini, Mohamed K. Fakhr

**Affiliations:** a Department of Biological Science, The University of Tulsa, Tulsa, Oklahoma, USA

## Abstract

Draft genome sequences of megaplasmid-bearing Streptomyces sp. strains BF-3 and 4F, isolated from the Great Salt Plains of Oklahoma, showed genome sizes of 7,950,134 and 7,550,992 bp, respectively. Both genomes revealed the presence of genes involved in osmoregulation and stress response, potentially helping their survival in such an extreme environment.

## GENOME ANNOUNCEMENT

Streptomyces is the most commonly known and widely studied genus of actinomycetes due to its extensive range of secondary metabolites that are important in medical, veterinary, industrial, and agricultural processes and, interestingly, carry linear chromosomes (1). While the majority of actinomycetes studied to date have been found in terrestrial environments, these organisms have been identified in a wide range of stressful settings and marine locations (2–4). One of the saline environments where actinomycetes have been identified is the Great Salt Plains of Oklahoma (5). We recently announced the whole-genome sequence of Bacillus velezensis strain SB1216, isolated from the Great Salt Plains of Oklahoma, which has a novel extracellular RNase with antitumor activity (6).

Here, we announce the draft whole-genome sequences of Streptomyces sp. strains BF-3 and 4F, previously isolated from the Great Salt Plains of Oklahoma, which showed the presence of approximately 200- and 400-kb megaplasmids, respectively (5, 7). The presence of megaplasmids was confirmed by pulsed field gel electrophoresis using S1 nuclease, as previously established in our laboratory to detect large plasmids (8). To our knowledge, these are the first draft whole-genome sequences to be published for a Streptomyces sp. isolated from the Great Salt Plains of Oklahoma.

DNA isolation was performed using a modified protocol for Gram-positive actinomycetes from a 72-h liquid culture of yeast extract-malt extract broth using a DNeasy blood and tissue kit (Qiagen, Inc., Valencia, CA, USA). Next-generation sequencing was performed after library preparation using the Nextera XT sample preparation kit (Illumina, Inc., San Diego, CA, USA). A MiSeq desktop sequencer was used for genome sequencing using an Illumina V2 reagent kit of 300 cycles with 100× coverage. The CLC Genomics Workbench version 7.5.1 was used to do the *de novo* assembly and analysis of the FASTQ output files of the short Illumina reads. Reference-guided trials for contig arrangement were done using the Microbial Genome Finishing Module version 1.4 (Qiagen, Inc.).

The draft genome of Streptomyces sp. strain BF-3 was 7,950,134 bp in size. Annotation according to the NCBI Prokaryotic Genome Annotation Pipeline revealed the presence of a total of 8,171 genes, in which 8,103 coding sequences (CDSs) and 7,411 coding genes were found. There were also 68 RNA genes and 692 pseudogenes present. The draft genome of Streptomyces sp. strain 4F was 7,550,992 bp in size with a total of 8,724 genes present, in which 8,655 CDSs and 7,758 coding genes were found. A total of 69 RNA genes and 897 pseudogenes were identified. The Streptomyces sp. strain BF-3 and 4F draft genomes showed the presence of genes coding for universal stress proteins, heavy metal resistance to cobalt, cadmium, zinc, arsenic, and tellurium and many genes involved in vitamin and antibiotic production and antibiotic resistance. Of special interest is the presence of genes coding for ectoine biosynthesis and regulation, coline and betaine uptake and biosynthesis, and heat and cold shock proteins, which help the organisms adapt to the harsh extreme environment of these salt plains.

### Accession number(s).

The whole-genome shotgun projects for Streptomyces sp. BF-3 and 4F have been deposited at DDBJ/ENA/GenBank under the accession numbers NACZ00000000 and NACY00000000, respectively. The versions described in this paper are the first versions.
